# A Spatio-Temporal Analysis of Matrix Protein and Nucleocapsid Trafficking during Vesicular Stomatitis Virus Uncoating

**DOI:** 10.1371/journal.ppat.1000994

**Published:** 2010-07-15

**Authors:** Chad E. Mire, Judith M. White, Michael A. Whitt

**Affiliations:** 1 Department of Molecular Sciences, University of Tennessee Health Science Center, Memphis, Tennessee, United States of America; 2 Department of Cell Biology, University of Virginia, Charlottesville, Virginia, United States of America; Wake Forest University School of Medicine, United States of America

## Abstract

To study VSV entry and the fate of incoming matrix (M) protein during virus uncoating we used recombinant viruses encoding M proteins with a C-terminal tetracysteine tag that could be fluorescently labeled using biarsenical (Lumio) compounds. We found that uncoating occurs early in the endocytic pathway and is inhibited by expression of dominant-negative (DN) Rab5, but is not inhibited by DN-Rab7 or DN-Rab11. Uncoating, as defined by the separation of nucleocapsids from M protein, occurred between 15 and 20 minutes post-entry and did not require microtubules or an intact actin cytoskeleton. Unexpectedly, the bulk of M protein remained associated with endosomal membranes after uncoating and was eventually trafficked to recycling endosomes. Another small, but significant fraction of M distributed to nuclear pore complexes, which was also not dependent on microtubules or polymerized actin. Quantification of fluorescence from high-resolution confocal micrographs indicated that after membrane fusion, M protein diffuses across the endosomal membrane with a concomitant increase in fluorescence from the Lumio label which occurred soon after the release of RNPs into the cytoplasm. These data support a new model for VSV uncoating in which RNPs are released from M which remains bound to the endosomal membrane rather than the dissociation of M protein from RNPs after release of the complex into the cytoplasm following membrane fusion.

## Author Summary

Vesicular stomatitis virus (VSV) is a prototypic enveloped virus that enters cells following endocytosis and a low pH-dependent membrane fusion event between the viral and endosomal membrane. To initiate a productive infection the viral nucleocapsid must dissociate from the matrix (M) protein, which underlies the viral membrane, in a process known as uncoating. The requirements for VSV uncoating are poorly understood. Here we used a virus containing fluorescent M protein to follow VSV uncoating in live cells. This analysis resulted in three new findings which provide for the first time a description of matrix and nucleocapsid trafficking during VSV uncoating. We found that most of the M protein remains bound to the endosomal membrane after virus-endosome fusion and that the nucleocapsid is released into the cytoplasm where replication occurs. While most of M remains membrane-bound, a small but detectable fraction is released during uncoating and is trafficked to nuclear pores. This has not been previously observed and may aid in shutting down host responses to infection. Collectively we provide the first spatio-temporal description of VSV uncoating by visualizing the uncoating process in live cells.

## Introduction

The entry of enveloped viruses that utilize the clathrin-dependent endocytic pathway involves attachment of virus to the cell surface and uptake of virions in coated vesicles that are transported to early or late endosomes. When virions reach a compartment in which the lumen has the appropriate pH there is an acid-induced fusion of the endosomal and viral membranes which results in virus uncoating and release of the genome into the cytoplasm [1], [2]. *Vesicular stomatitis virus* (VSV), a prototypic enveloped, nonsegmented, negative-strand RNA virus in the *Rhabdoviridae* family enters host cells through the clathrin- and pH-dependent endocytic pathway [3], [4], [5], [6]. The genome of VSV encodes five major viral proteins: the nucleocapsid protein (N), the phosphoprotein (P), the matrix protein (M), the glycoprotein (G), and the large polymerase protein (L). The viral genome is encapsidated by the N protein and associates with the viral RNA-dependent RNA polymerase (RdRp), which consists of a complex of the L and P proteins. The N-RNA-RdRp collectively forms the ribonucleoprotein (RNP) complex. The M protein within virions is associated with RNPs in structures called *skeletons* [7], [8]. Recently, cryo-EM imaging of intact VSV particles showed that the RNP skeleton consists of a compact left-handed helix bounded by an outer layer of M protein which anchors the RNP to the viral membrane [9]. Protruding from the virion surface are glycoprotein spikes consisting of G protein trimers. G protein is responsible for attachment of virions to cells and fusion of the endosomal and viral membranes, which results in the transfer of the RNP into the cytoplasm where VSV replication occurs [3]. Early models of VSV uncoating proposed that either directly after or concomitant with membrane fusion, M protein dissociates from RNPs, which results in decondensation of the *skeleton* [7], [8] and completes virus uncoating [10]. More recently, it was proposed that VSV initially fuses with vesicles found within multivesicular bodies and that the release of nucleocapsids into the cytoplasm occurs after transport to late endosomes, which requires a back-fusion event [11], and that Tsg101 controls the endosome-to-cytosol release of nucleocapsids [12]. After uncoating, the decondensed RNP serves as a template for transcription of viral mRNAs by the packaged RNA-dependent RNA polymerase.

VSV uncoating, defined as the dissociation of M from RNPs, is an essential step which is required for a productive infection [13]. Without uncoating it is thought that transcription of viral mRNAs will not occur since it has been shown that transcription from RNPs is inhibited by M protein [14], [15], [16]. This essential step is also shared by another enveloped negative-strand virus, *influenza virus*, which requires the release of its matrix protein (M1) for a productive infection [17], [18]. To better understand the requirements for VSV uncoating, we generated recombinant VSV (rVSV) which encoded M proteins that had tetracysteine (-CCRECC-) Lumio tags at either the N- or C-terminus, and recovered infectious virus [19]. Similar rVSVs have been described by others [20] and have been used to study VSV entry and assembly. Previously, using rVSV containing M protein labeled with Lumio Green (rVSV-MLG), we found that during entry the interior of VSV virions become acidified and that acidification required G protein [19]. Furthermore, we obtained evidence that virion acidification enhanced M protein release and proposed a model in which G protein-induced acidification facilitates VSV uncoating by causing subtle conformational changes in M protein which results in the dissociation of M from RNPs.

In this report we examine the kinetics of VSV uncoating and the fate of virion-associated M protein and RNPs during VSV entry and after uncoating using rVSV-MLG. We show that after fusion of the viral membrane with endosomes, the bulk of M protein remained associated with vesicular structures and eventually colocalized with markers for recycling endosomes. Although most of M remained bound to endosomal membranes, a small, but significant fraction of M protein was released and localized to the nuclear envelope. The delivery of M protein to the nuclear envelope was not dependent on microtubules or an intact actin cytoskeleton. Using confocal microscopy we observed that RNPs entered the cytoplasm and physically separated from M protein between 15 and 20 minutes post-entry and that the release of RNPs into the cytoplasm was also not dependent on microtubules or intact microfilaments. Following RNP release, the membrane-bound M protein diffuses across the endosomal membrane with a concomitant increase in MLG fluorescence. Collectively, these data provide strong evidence that VSV uncoating involves the direct release of RNPs from membrane-associated M protein into the cytoplasm where viral transcription can take place to initiate a productive infection.

## Results

### Productive infection requires Rab5, but not Rab7 or Rab11

Recently, using rVSV containing Lumio-Green labeled M protein (rVSV-MLG), we reported that very soon after endocytosis the interior of VSV virions become acidified resulting in a decrease in input MLG fluorescence [19]. This is followed by a recovery of fluorescence to the original input levels at approximately 10 minutes post-entry, which we proposed is due to exposure of MLG to the neutral pH of the cytoplasm and therefore is a marker for VSV membrane fusion and the initiation of virus uncoating [19]. These results are consistent with models in which the release of VSV RNPs occurs from early endosomes.

**Figure 1:**
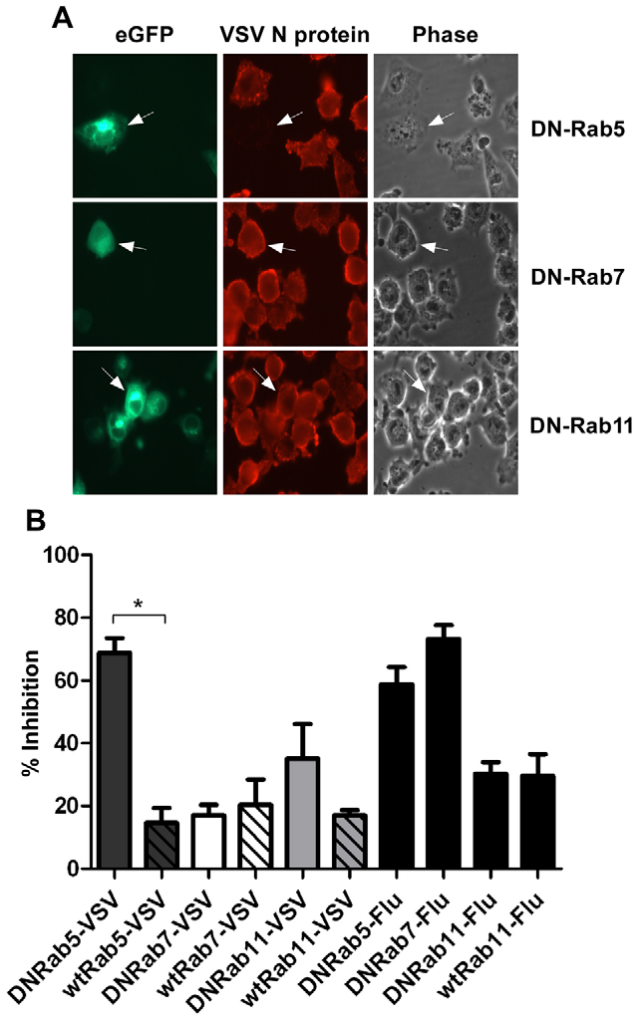
Effect of DN-Rab expression on VSV infection. Cells were transfected with plasmids expressing DN- or wt-Rab-eGFP proteins and infected with rVSV-wt or influenza virus at 18 hours post-transfection. Cells were fixed, permeabilized and stained for VSV N protein or influenza NP at 8 hr post-infection. (A) Fluorescence and brightfield micrographs showing the effect of DN-Rab5 (top panels), DN-Rab7 (middle panels), and DN-Rab11 (bottom panels) on VSV infection. Arrows mark cells expressing Rab-eGFP proteins. (B) Inhibition of virus infection was quantified by determining the number of Rab-positive cells that were also infected by VSV or influenza virus. Statistical significance for effect on VSV infection was determined using Student's t-test (* =  p < 0.002) from 152 cells (Rab5), 153 cells (Rab7), or 156 cells (Rab11).

To further define the intracellular location of VSV uncoating we examined the effect of dominant-negative (DN) Rab proteins on rVSV-wt infection. Rab5, 7 and 11 are GTPases that are required for the movement of endocytosed cargo to early (Rab5), late (Rab7), or recycling (Rab11) endosomes [21], [22], [23]. Expression of DN-Rab5 prevents fusion of endocytic vesicles with early endosomes, while DN-Rab7 and 11 prevent movement from early to late, or to recycling endosomes, respectively. BHK-21 cells were transfected with plasmids encoding eGFP-tagged DN-Rabs and then the cells were infected with rVSV-wt 18 h post-transfection. The cells were fixed 8 hours later, permeabilized, and stained for VSV nucleocapsid (N) protein using an N-specific monoclonal antibody (mAb-10G4; [24]) conjugated to Alexa Fluor-568. As shown in Fig. 1, most cells expressing DN-Rab7 and DN-Rab11 were infected, but cells expressing DN-Rab5 were not and infection was inhibited by ∼70%. Expression of the wt-Rab proteins had only minimal effects on VSV infection (Fig. 1B). These data support previous reports which showed VSV could infect cells expressing DN-Rab7, but not cells expressing DN-Rab5 [4], [5]. Inhibition of VSV infection by DN-Rab5 was highly significant (p = 0.002), but the small inhibition observed with DN-Rab11 was not (p = 0.1549). To ensure our DN-Rab7 construct was functional we examined the effect of the DN-Rabs on influenza A virus infection. As shown previously [5], DN-Rab5 and 7 inhibited influenza virus infection (Fig. 1B), while DN-Rab11 did not. Collectively, these data indicate that the majority of VSV virions fuse and uncoat after delivery to early endosomes and that VSV infection does not require transfer to late endosomes.

### Live-cell entry of rVSV-MLG

**Figure 2:**
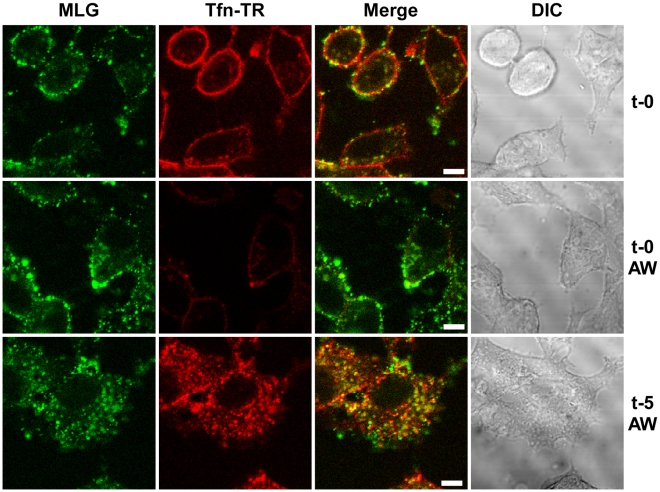
Endocytosis of surface bound Tfn-TR and rVSV-MLG. rVSV-MLG and Tfn-TR were bound to cells at 4°C for 90 minutes and the cells were either (top panels) fixed immediately with ice-cold 3% paraformaldehyde (t-0), or (middle panels) treated with acidic desferrioxamine to strip Tfn-TR from the cell surface (t-0, AW) and then fixed, or (bottom panels) warmed by replacing the inoculum with 37°C media for 2 minutes followed by quickly cooling with the addition of ice-cold PBS, and then acid-desferrioxamine washed on ice. After the acid wash the cells were warmed to 37°C, the cells were incubated for an additional 3 minutes (t-5, AW) and then examined without fixation by LSCM on a heated stage. Bars  =  5 µm. DIC  =  differential interference contrast.

**Figure 3:**
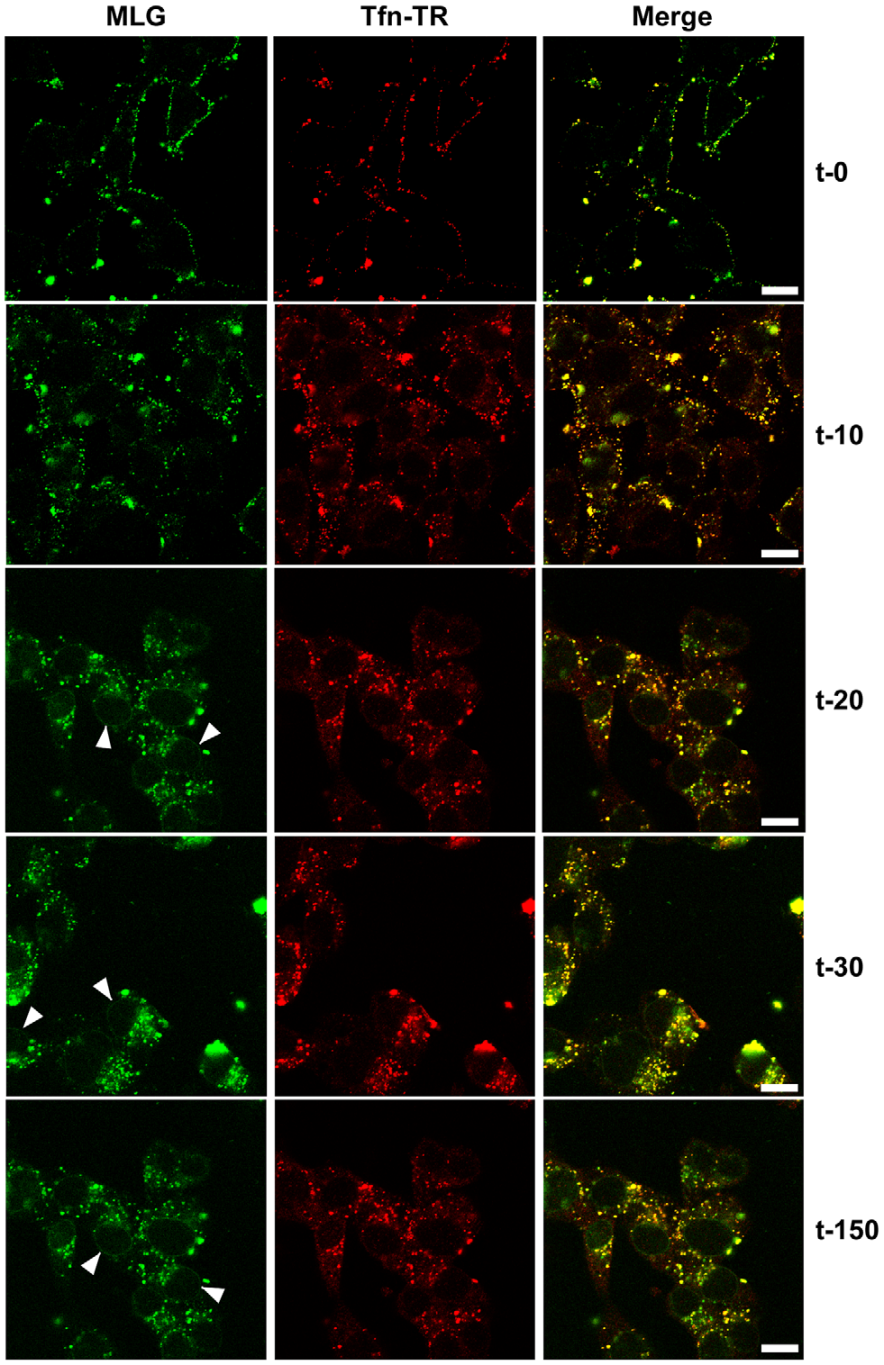
Uncoating and distribution of M protein in live cells. Cells were inoculated with rVSV-MLG and Tfn-TR at 4°C for 90 minutes and either fixed immediately with ice-cold 3% paraformaldehyde (t-0) or were quickly warmed by the addition of 37°C media and examined without fixation by LSCM on a heated stage at the indicated times using separate 35mm glass bottomed dishes for each time point to reduce signal loss by photobleaching. Bars  =  10 µm. Arrowheads indicate MLG localization to NPCs.

**Figure 4:**
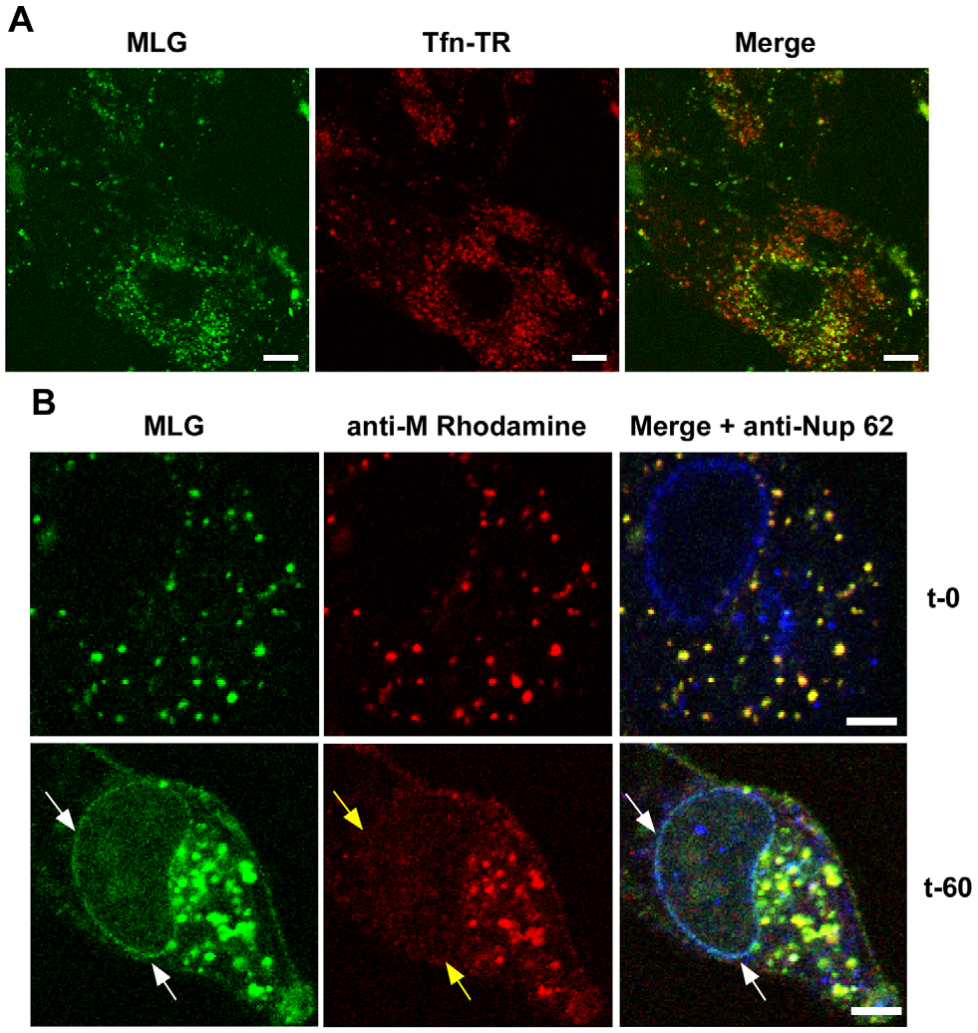
Localization of MLG at the nuclear envelope. (A) BHK cells were pre-treated with bafilomycin A1 (Baf) and then rVSV-MLG and Tfn-TR were bound to cells in the cold in media containing Baf for 90 minutes. The inoculum was removed and the cells were incubated for 150 minutes at 37°C in media containing Baf. Cells were imaged by LSCM as described in Fig. 3. Bars  =  10 µm. (B) Cells were infected with rVSV-MLG in the presence of NH_4_Cl for 90 minutes to allow accumulation of virus in endosomes and then entry was initiated by incubation in media without NH_4_Cl, but containing cycloheximide to prevent new viral protein synthesis. Cells were fixed at t-0 (top panels) and t-60 (bottom panels), stained for M protein using a rhodamine conjugated anti-M mAb (red) and a Nup62 antibody labeled with Alexa Fluor-647 (blue), and examined by LSCM. White arrows indicate the localization of MLG at the nuclear envelope. Note the M mAb does not detect M protein localized to the nuclear envelope (middle panels, yellow arrows). Bars  =  2 µm.

**Figure 5:**
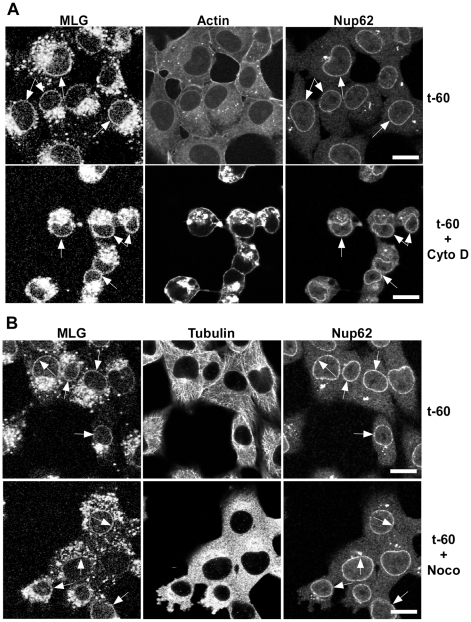
Distribution of MLG in the presence of cytochalasin D or nocodazole. BHK cells were infected with rVSV-MLG in the presence of NH_4_Cl as described for Fig. 4B. Cytoskeletal inhibitors were added for 30 minutes before NH_4_Cl washout and the inhibitors remained in the post-washout media until the cells were fixed 60 minutes later. Distribution of MLG, Nup62 and actin (A) or tubulin (B) either without (top panels) or with (bottom panels) cytoskeletal inhibitors. Arrows indicate nuclear envelope localization. The brightness levels were adjusted for all the images using Canvas 11 software to maximize the MLG signal after conversion to grayscale. Bars  =  10 µm.

To study VSV uncoating and the fate of incoming M protein, rVSV-MLG and transferrin-Texas Red (Tfn-TR) were adsorbed to cells for 90 minutes at 4°C to prevent endocytosis and then entry was initiated by replacing the inoculum with media pre-warmed to 37°C. The Tfn-TR was used as a surface (t-0) and endosomal (post t-0) marker for most of the studies described below. To differentiate surface-bound from endocytosed Tfn-TR, the cells were treated with acidic buffer containing an iron chelating agent, which removes transferrin bound to transferrin receptor on the cell surface. Acid washing (AW) of cells prior to the addition of warm media and the initiation of endocytosis resulted in the loss of the Tfn-TR signal (Fig. 2, t-0 AW), but had no effect on bound virus. Five minutes after transferring the cells to 37°C virtually all of the Tfn-TR was endocytosed (Fig. 2, t-5 AW). Based on colocalization of Tfn-TR and MLG fluorescence, the vast majority of rVSV-MLG was also endocytosed, which is consistent with two recent reports showing that VSV is rapidly internalized [4], [6]. Because of concerns that acid treatment may induce membrane fusion of surface bound virus, all of the subsequent studies were performed without an acid wash step.

### The bulk of M protein remains associated with transferrin-positive endosomes

To examine the kinetics of virus entry and to follow the trafficking of M protein, rVSV-MLG and Tfn-TR were bound to cells in the cold as described in Fig. 2, and after the addition of warm media the cells were incubated for the specified times and examined by laser scanning confocal microscopy (LSCM; Fig. 3). At t-0, rVSV-MLG and Tfn-TR decorated the cell surface and showed some colocalization. After 5 min (not shown) both markers were internalized and most of the rVSV-MLG virions were in transferrin-positive endosomes. Between 10 to 30 minutes, endosomes containing Tfn-TR and MLG moved from the cell periphery towards the cell interior. Notably, the bulk of MLG remained associated with Tfn-TR-positive endosomes until the last time point examined (t-150).

### Delivery of M to the nuclear envelope

In addition to the endosomal-associated fraction a small amount of MLG began to accumulate on what appeared to be the nuclear envelope beginning 20 minutes post entry (Fig. 3, arrowheads). Association of MLG with this structure continued to increase up to the last time point examined. At no time did we observe Tfn-TR associated with the nuclear envelope suggesting this was not due to membrane recycling to the endoplasmic reticulum surrounding the nucleus.

To determine if trafficking of MLG to the nuclear envelope required membrane fusion and virus uncoating rVSV-MLG and Tfn-TR were bound to cells that had been pretreated with bafilomycin A1, which inhibits endosome acidification and prevents VSV infection, and examined by LSCM at various times as described for Fig. 3. At no time did we observe MLG associating with the nuclear envelope in cells treated with bafilomycin A1 (Fig. 4A). To determine if ongoing protein synthesis was required we synchronized fusion of the viral and endosomal membrane using ammonium chloride in the presence of cycloheximide. Ammonium chloride (NH_4_Cl) is a lysosomotropic agent that prevents acidification of endosomes and thereby inhibits fusion and uncoating of endocytosed virions [10], [25], but unlike bafilomycin A1, NH_4_Cl can be easily washed out of cells allowing rapid re-acidification of endocytic compartments. Following entry in the presence of NH_4_Cl, the inoculum and NH_4_Cl were removed, the cells were washed 4 times, and media containing cycloheximide was added. Cells were fixed either immediately or 60 minutes after NH_4_Cl removal. The cells were then permeabilized and stained with anti-Nup62 antibody, which binds to the nuclear pore complex (NPC) on the nuclear envelope, and with an anti-M mAb (23H12; [24]) and examined by LSCM (Fig. 4B). At t-0, rVSV-MLG particles were in small vesicular structures (endosomes) and no M protein was detected at the nuclear envelope (Fig. 4B, top panels). Similar to the results shown in Fig. 3, at t-60 the bulk of MLG was found in large, mostly perinuclear endosomes and a small but significant amount of MLG was localized to the nuclear envelope (Fig. 4B, t-60 arrows). These data indicate that while most of the incoming (e.g. virion-associated) M protein remains associated with Tfn-positive endosomes, a small amount of M trafficks to the NPC after uncoating. As reported by others, the M mAb (23H12) did not detect M protein bound to nuclear envelope (Fig. 4B, yellow arrows), which explains why earlier studies examining VSV uncoating [10] did not observe delivery of M protein to the NPC. The epitope recognized by the 23H12 M mAb overlaps the region of M that binds to the nuclear shuttling protein RaeI [26], [27], suggesting that the fraction of MLG which associates with the nuclear envelope is likely complexed with RaeI.

### Trafficking of MLG in the presence of cytoskeletal inhibitors

To determine if microtubules or microfilaments are required for the delivery of MLG to the nuclear envelope cells were inoculated with rVSV-MLG in the presence of NH_4_Cl for 60 minutes and then the cytoskeletal inhibitors cytochalasin D or nocodazole were added for 30 minutes. The NH_4_Cl was removed by washing the cells with media containing cycloheximide either with or without cytoskeletal inhibitors and incubated for an additional 60 minutes. The cells were then fixed and stained for filamentous actin using Texas Red-phalloidin (Fig. 5A), or for tubulin (Fig. 5B). Either in the absence (top panels) or presence of the inhibitors (bottom panels), MLG was found in association with endosomes and at the nuclear envelope (arrows). These results indicate that delivery of MLG to the nuclear envelope does not require an intact actin cytoskeleton or microtubules.

### Colocalization of MLG and markers of recycling endosomes

**Figure 6:**
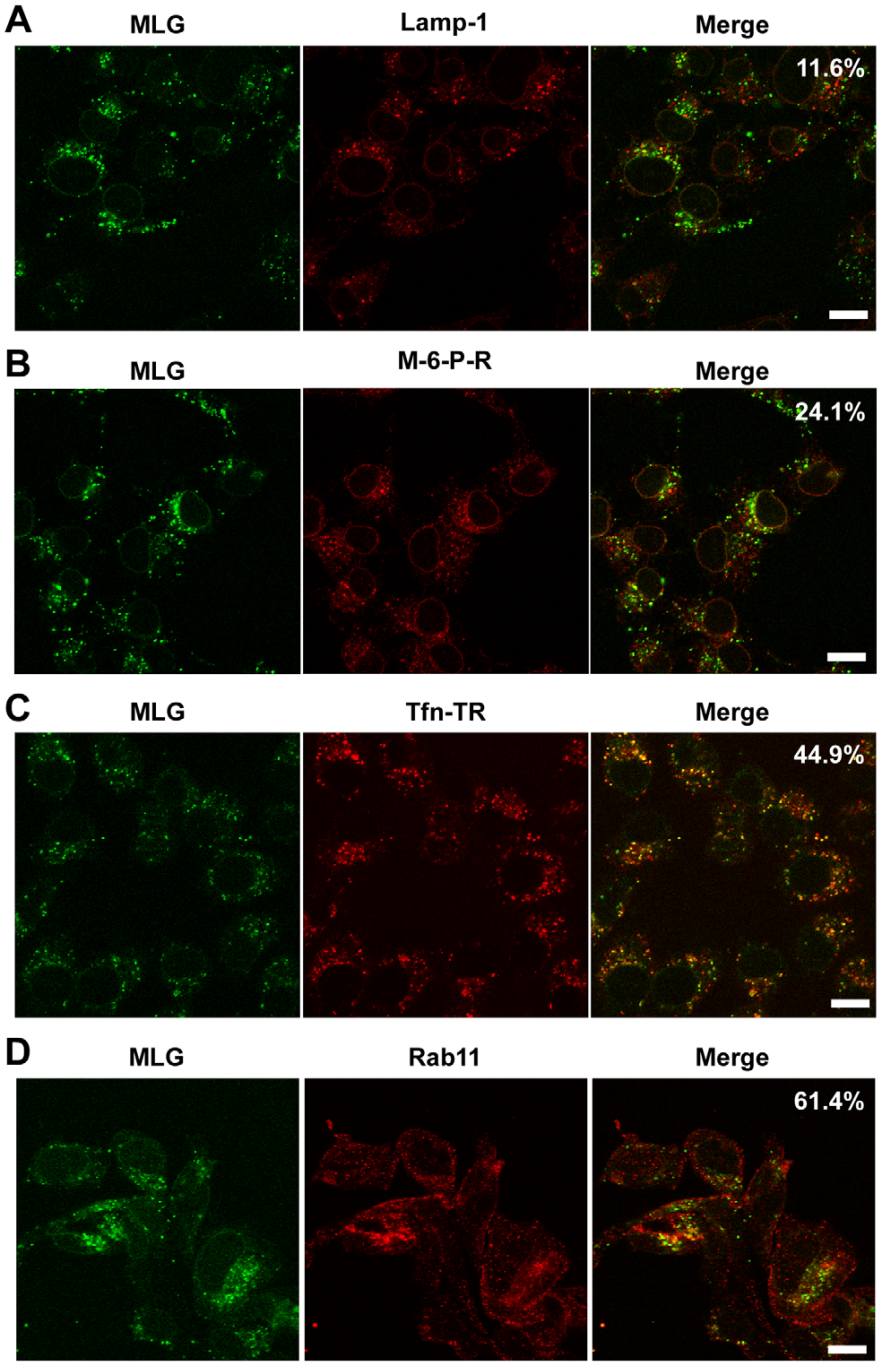
Colocalization of MLG with markers for recycling endosomes. A synchronized fusion assay was performed as described for Fig. 4B, the cells were fixed at t-60, stained for (A) LAMP-1, (B) mannose-6-phosphate receptor, (C) Tfn-TR, or (D) Rab11 and then examined by LSCM (Bars  =  10 µm). Percent colocalization of MLG with the indicated marker from approximately 20 individual cells from two independent experiments is shown in the upper right-hand corner of the merged images.

The images in Fig. 3 show that the bulk of MLG remains associated with Tfn-positive endosomes for at least 150 min after entry. To determine the identity of these perinuclear structures we used markers that corresponded to lysosomes (LAMP-1), late endosomes/lysosomes (Mannose-6-Phosphate receptor, M-6-P-R), or recycling endosomes (Tfn-TR and Rab11). rVSV-MLG was endocytosed in the presence of NH_4_Cl as described for Fig. 4, the inoculum and NH_4_Cl were removed and the cells were incubated for an additional 60 min and then stained using antibodies to the various endosomal markers, or visualized directly for Tfn-TR. Quantification of MLG colocalization with the various markers indicated that most of incoming M protein remains associated with membranes that mature into recycling endosomes (Fig. 6C and D), while only some MLG is delivered to late endosomes (Fig. 6B) and lysosomes (Fig. 6A).

### Separation of RNPs from MLG correlates with productive infection and does not require microtubules

**Figure 7:**
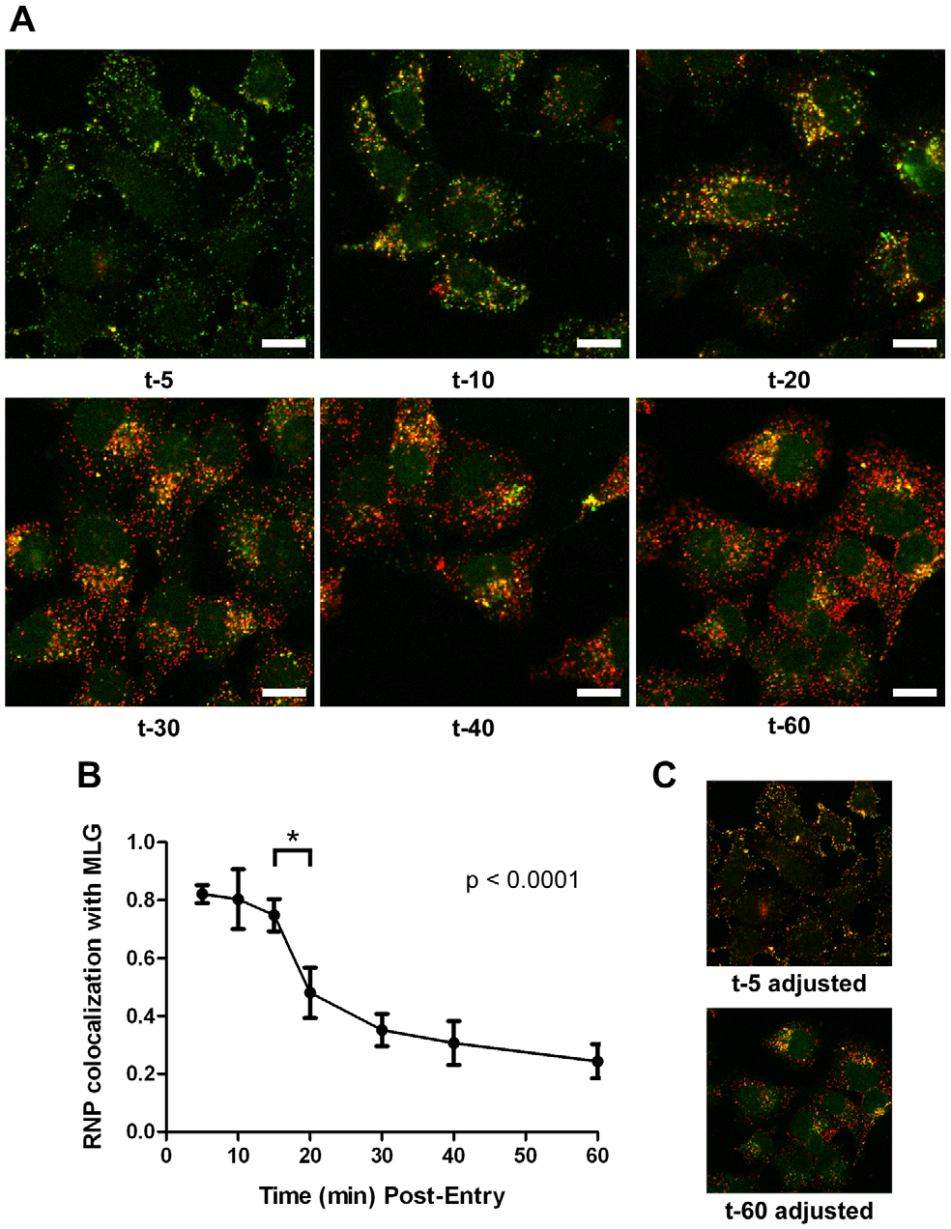
Kinetics of RNP separation from MLG-endosomes. (A) rVSV-MLG was bound to cells in the cold as described for Fig. 3 except Tfn-TR was omitted. Cells were fixed at the times indicated, permeabilized and RNPs were detected by staining for N protein using an N mAb conjugated to Alexa Fluor-568. Images were collected by LSCM using identical detector, offset, and gain settings. Bars  =  10 µm. (B) Colocalization of RNPs with MLG from the images in (A) as a function of time post-entry. Significance was determined using Student's t-test (n  =  9 cells). (C) The red and green channel levels for the t-5 and t-60 images from (A) were adjusted in Adobe Photoshop to give similar red and green intensities, which shows better the colocalization of RNPs with MLG detected by the LSM software for the t-5 time point.

**Figure 8:**
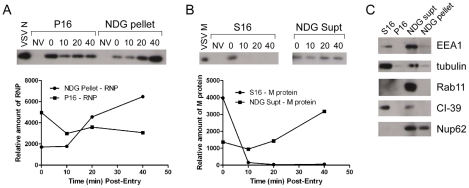
Cell fractionation and analysis of the distribution of RNPs and M protein during virus entry. rVSV-wt was bound to cells in the cold for 90 minutes in the presence of cycloheximide to prevent new protein synthesis, the inoculum was removed and the cells were washed and then either removed from the dish immediately (t-0) or warmed to 37°C and then harvested for fractionation at the times indicated. Fractions were subjected to immunoblot analysis using polyclonal anti-VSV sera. Relevant regions of the immunoblot are shown. NV (no virus) indicates cells that were mock infected and harvested at t-0. Graphs below the blots show quantification of N or M protein in each fraction. (A) N protein detected in the P16 (plasma membrane-associated virions) and NDG pellet (detergent-resistant nucleocapsids) fractions. Times post-entry are shown above the immunoblot. VSV N is a lane containing purified virus. (B) M protein detected in the S16 (plasma membrane and mitochondrial membrane) and NDG supernatant (endosomal and nuclear membrane) fraction. NV (no virus) mock infected cells. VSV M is the lane containing purified virus. (C) The four fractions probed with antibodies to the indicated cellular markers.

It is not known if VSV RNPs are released into the cytoplasm, or if they remain associated with membranes after uncoating *in vivo*. To further define the site of virus uncoating we asked whether we could detect nucleocapsids physically separating from MLG after endocytosis. Cells were inoculated with rVSV-MLG as described in Fig. 3, except that Tfn-TR was not included, and entry was initiated after transfer to 37°C. Cells were fixed and RNPs were detected by staining for N protein at the times indicated. At t-5, the majority of RNPs colocalized with MLG (Fig. 7A and C). Beginning approximately 10 minutes post-entry RNPs could be seen separating from MLG-endosomes. The largest change in RNP-MLG colocalization occurred between 15 and 20 minutes; quantification of the fraction of RNPs that colocalized with MLG decreased from approximately 80% at t-5 through t-15 to less than 50% at t-20 (Fig. 7B). We also examined RNP-M separation biochemically using a cell fractionation protocol to determine if the results seen by fluorescence confocal microscopy could be reproduced by an independent method. As shown in Fig. 8A, RNPs redistributed from intact virions at t-0 to a cytoplasmic fraction between 10 and 20 minutes post-entry, similar to the time when RNPs dissociated from MLG in Fig. 7. Likewise, M protein redistributed to a fraction that was enriched in endosomal markers beginning at 10 minutes and continuing until the end of the experiment (Fig. 8B). Thus, the distribution of RNPs and M protein correlated well when using either confocal microscopy or a cell fractionation assay.

**Figure 9:**
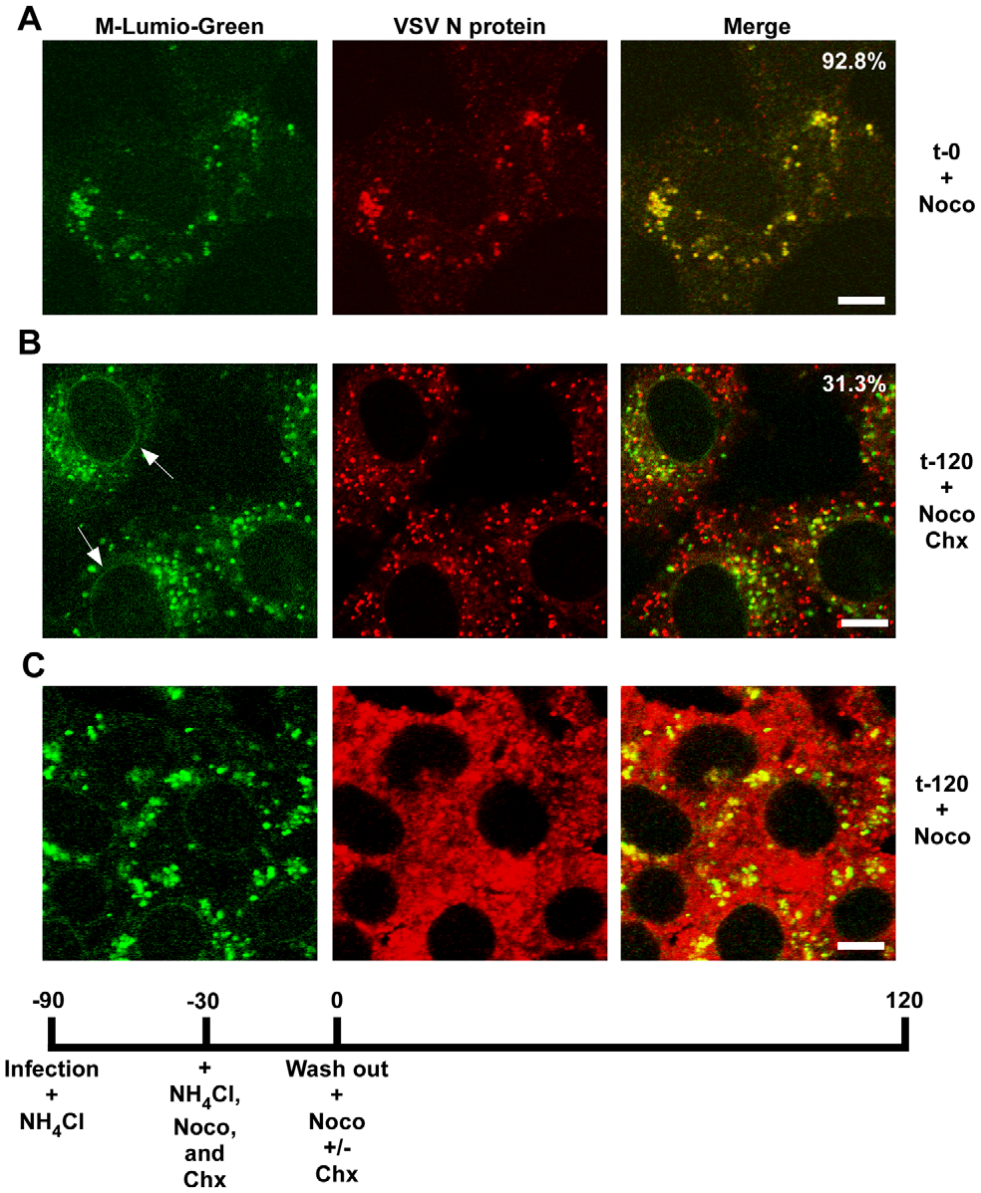
Release of RNPs from endosomes and viral protein synthesis in the presence of nocodazole. Confocal images of cells inoculated with rVSV-MLG at t-0 (A) and t-120 (B and C) post-NH_4_Cl washout in the presence of cycloheximide and nocodazole (B), or just nocodazole (C), and stained for VSV N protein using N mAb conjugated to Alexa Fluor-568. Quantification of the amount of colocalization was determined for 50 individual cells using the colocalization function in the LSM software version 3.2. Percent of N protein that colocalized with MLG is indicated for t-0 and t-120 (n  =  50 cells). Bars  =  5 µm.

To determine if RNP separation required microtubules rVSV-MLG was endocytosed in the presence of NH_4_Cl for 1 hour and then nocodazole and cycloheximide were added for 30 minutes to depolymerize microtubules and stop protein synthesis. The cells were either fixed immediately (Fig. 9A) or were incubated in the presence of both inhibitors, or with nocodazole only, for an additional 2 hours. Quantification of MLG and N protein colocalization showed that at t-0, 92.8% (+/−3.4%, n  =  50 cells) of RNPs colocalized with MLG, whereas by t-120 (Fig. 9B) most of the nucleocapsids had separated from M protein as indicated by only 31.3% (+/−8.2%, n  =  50 cells) of RNP-MLG colocalization. Similar to that observed in Figs. 3, 4 and 5, there was very distinct MLG fluorescence associated with the nuclear envelope at t-120, but importantly there was no N protein staining of the nuclear membrane. This indicates that M protein localizes to the nuclear envelope alone and not in association with RNPs. As previously reported by others [28], we also observed that VSV transcription and viral protein synthesis can occur following infection of cells that lack an organized microtubule network (Fig. 9C), as indicated by the extensive N protein staining throughout the cytoplasm which represents new N protein synthesis. These results also confirm that the N protein staining seen in Fig. 9B represents incoming RNPs, and is not from new N protein synthesis.

### Spatial distribution of membrane bound-MLG during VSV uncoating

When fusion between the virus and endosomal membranes occurs, an asymmetry in the endosomal membrane is generated by addition of the viral membrane to the endosomal membrane at the site of membrane fusion. Based on the tight association of MLG with the viral membrane it should be possible to visualize the virus-endosome fusion event using MLG as a marker.

**Figure 10:**
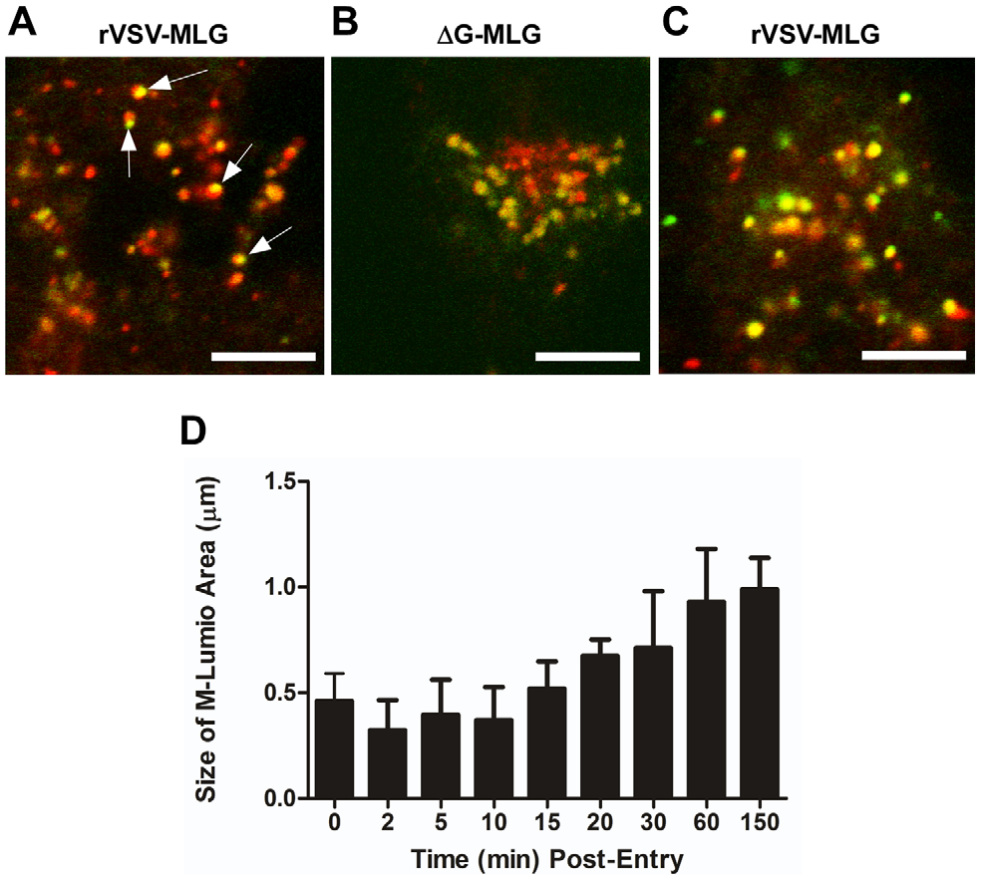
Spatio-temporal distribution of MLG during virus entry and uncoating. A synchronized entry assay was performed as described for Fig. 4B using either rVSV-MLG (A) or “bald” ΔG-MLG (B) and the cells were imaged 15 minutes after addition of 37°C media. A region having a high concentration of endosomes was magnified to 500× using the “crop” function of the LSM software to show intracellular vesicles containing MLG and Tfn-TR. Arrows indicate an asymmetric distribution of MLG within Tfn-TR positive endosomes in rVSV-MLG infected cells. Bars  =  5 µm. (C) MLG distribution 150 minutes post-entry. (D) The sizes of the MLG “patch” found on Tfn-TR endosomes (n  =  30 endosomes) from a live cell experiment similar to that shown in Fig. 3 were measured using the Profile function of the Zeiss LSM510 software, version 3.2 and plotted as a function of time post-entry.

To examine the spatial distribution of MLG during virus entry and uncoating we analyzed high magnification confocal images of endosomes from an experiment similar to that described in Fig. 3. For this analysis we used a 100X-1.4 N.A. apochromat objective and digitally magnified the image 5× prior to capture using 1024×1024 pixel resolution. Figure 10A shows an example of an image collected 15 minutes post-entry which clearly shows an asymmetric distribution of MLG relative to Tfn-TR within an endosome. To determine if this asymmetry required membrane fusion we produced MLG virus particles lacking G protein (ΔG-MLG). We have shown previously that “bald” ΔG-VSV is able to bind and enter cells, albeit somewhat less efficiently than wt-VSV, and that the virus is non-infectious due to the lack of G protein [29]. In contrast to infectious rVSV-MLG, we found that MLG from ΔG-MLG particles did not acquire an asymmetrical distribution (Fig. 10B), presumably because the particle remained within the lumen of the endosome. As might be expected, we also did not observe MLG from ΔG-MLG particles associating with the nuclear envelope at any time point (data not shown).

In addition to observing MLG at the site of membrane fusion, we followed the fate of MLG post-fusion. We found that by 20 to 30 minutes post-entry the asymmetry began to disappear and the size of the MLG-positive membrane area increased, reaching a plateau after approximately 60 minutes (Fig. 10C and D). These data suggest that after binding of virus to cells on ice, virus-endosome fusion occurs between 10 and 15 minutes after warming cells to 37°C. The data also suggest that as M protein is exposed to the cytoplasm following membrane fusion it begins to diffuse across the endosomal membrane where it becomes evenly distributed as the lipids of the viral and endosomal membranes continue to mix. The kinetics of the increase in MLG surface area correlated with our previously published results [19] showing that after an initial low pH-induced decrease in MLG fluorescence, there was a steady increase in fluorescence up to approximately 60 minutes post-entry. Based on the results shown in Fig. 10D, the increase in fluorescence likely represents reduced packing of MLG as it transitions from the ordered helical lattice found within virions [9], to a more diffuse form found after uncoating has occurred. This change in fluorescence and movement of MLG across the endosomal membrane also correlates temporally with the reduction in colocalization of RNPs with MLG, which represents the release of RNPs into the cytoplasm seen in Fig. 7.

## Discussion

To study VSV entry we generated rVSVs encoding fluorescently tagged M proteins (rVSV-MLG) using Lumio technology and recently reported that very soon after initiation of rVSV-MLG entry there is a transient decrease in MLG fluorescence, which we suggested is due to acidification of the virion interior [19]. Between 5 and 10 minutes post-entry MLG fluorescence recovers to input levels, which is followed by a steady increase in fluorescence that plateaus between 60 and 150 minutes post-entry. In this report we extend these studies by examining the intracellular distribution of M protein during and after VSV uncoating and show that the bulk of M protein remains bound to endosomes while RNPs are released into the cytosol. We confirmed previous reports by others [4], [5], that VSV infection requires Rab5, but not Rab7, and extended these studies by showing that DN-Rab11 also did not inhibit VSV infection (Fig. 1). Our studies also provide the first kinetic description of VSV uncoating with respect to the release of RNPs following membrane fusion and the spatial relationship of M protein and RNPs during the uncoating process.

### Finding 1: M remains associated with endosomes after uncoating

An important new finding from these studies was that most of the incoming M protein remained associated with an endosomal membrane at both early and at late times after entry. This was seen both in cells that had virus bound to the cell surface in the cold, as well as in cells where virus fusion was synchronized by NH_4_Cl washout. By quantifying the amount of N protein that had separated from MLG endosomes (Fig. 7), it appears that greater than 75% of the virus had fused, therefore only a small fraction represents unfused virus. Thus, most of the incoming virus enters cells in a productive manner and the bulk of M protein remains bound to the endosomal membrane. The topology of the viral membrane after fusion would predict that the endosomal-associated M would be exposed to the cytoplasm; therefore the binding of M to the endosomal membrane must be quite strong.

### Finding 2: M trafficking to NPCs

As the infection progressed, e.g. 60 to 150 minutes post-entry, M protein was observed in large perinuclear structures, which stained for markers of recycling endosomes. Following uncoating a smaller proportion of M was apparently released and rapidly localized to the nuclear envelope. These observations correlate nicely with an earlier study which showed using a biochemical assay that ∼15% of input M was released into the cytosol after entry [10]; however, in that study the distinct nuclear envelope localization was not observed. Our results support the finding that only a fraction of the incoming M protein is released in a soluble form after uncoating. However, this small amount of M colocalized with Nup62, suggesting that this soluble fraction binds to nuclear pore complexes (NPCs). These data are consistent with reports that GFP-M fusion proteins can associate with NPCs [27]. Based on our finding that M protein from incoming virus particles also binds to NPCs, we propose that upon VSV entry, a small amount of M protein is released after uncoating and that this fraction associates with NPCs where it may block nucleocytoplasmic transport [30], thus representing an early effort by the virus to mute the innate immune response even before viral protein synthesis has begun.

The mechanism by which M is trafficked to the NPCs is less clear. We observed localization of MLG on the nuclear envelope in the absence of polymerized actin (Fig. 5A) or tubulin (Fig. 5B). This was unexpected since early studies indicated that M protein binds to the cytoskeleton [31], [32] and it was assumed that M trafficking to the nuclear envelope involves microfilaments or microtubules. Alternatively, trafficking of M to the NPC may involve M binding to RaeI. It is known that binding of M to RaeI results in inhibition of mRNA nucleo-cytoplasmic transport and that RaeI binds to microtubules in mitotic spindle complexes [33], [34]. Therefore, M could bind to RaeI in the cytoplasm and be delivered to the NPCs; however, we observed M trafficking to the nuclear envelope in the presence of nocodazole, suggesting that localization to the NPC likely occurs by a different mechanism.

Our results also shed new light on the mechanism of VSV entry and uncoating. There are currently two models proposed for how VSV nucleocapsids are delivered to the cytoplasm. Both models begin with the well-accepted paradigm that VSV particles are endocytosed via the clathrin-dependent pathway and are delivered to early endosomes. In the traditional model of VSV entry, once the pH of the early endosomal lumen becomes acidified to ∼pH 6.3, G protein undergoes conformational changes which induce fusion of the viral envelope with the endosomal membrane and the RNP is exposed to the cytoplasm where primary transcription occurs. This model has gained support through the use of siRNAs to knock-down essential Rab proteins required for maturation of early to late endosomes [5], [21] and by use of specific inhibitors and TIRF microscopy [4]. A second model was recently proposed in which fusion occurs early, but RNPs are not released immediately and instead are trafficked in multi-vesicular bodies to late endosomes where a “back-fusion” event occurs which releases RNPs into the cytoplasm [11], [12]. Our data support the traditional model (at least for the majority of particles) in which VSV virions fuse early in the endocytic pathway and RNPs are released, with the unexpected observation that a significant pool of M protein remains associated with the endosomal membrane long after RNP release has occurred.

### Finding 3: RNPs are released from endosome-bound M protein

**Figure 11:**
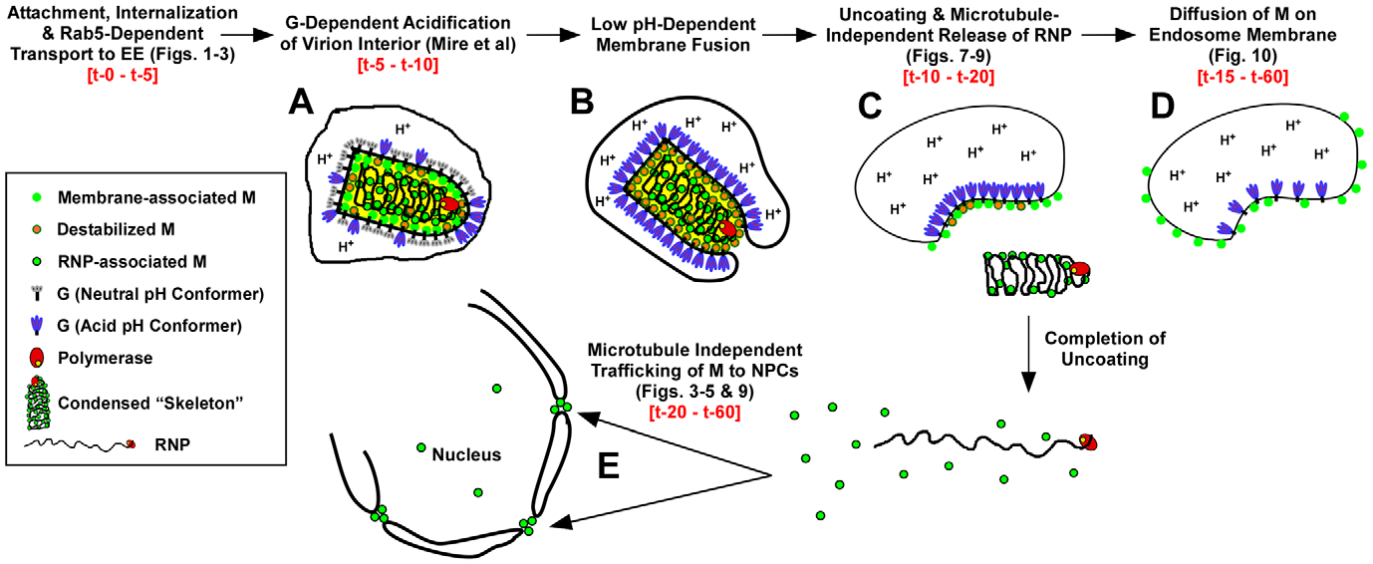
Time-line of VSV entry and uncoating events. A model for VSV entry and uncoating based on the spatio-temporal imaging analysis of MLG and RNP from rVSV-MLG. Relevant figures from the analyses are shown in parentheses. Observed times of the events are shown in red-colored font. In the model, virions attach to the cell surface, are endocytosed and trafficked to early endosomes in a Rab5-dependent manner where low pH induces conformational changes G resulting in acidification of the virion interior (A) and subsequent fusion of the viral and endosomal membrane (B). Acidification of the virion interior has an effect on the M protein to destabilize interactions between membrane-associated M (green circles with orange borders) and the condensed RNP, which results in release of the RNP into the cytoplasm after membrane fusion (C). The bulk of M remains associated with endosomes, but begins to diffuse across the endosomal membrane after RNP release (D). A smaller fraction of M is released into the cytosol and reaches the NPCs in a microtubule and actin independent manner (E).

The use of fluorescently labeled M and the direct visualization of M distribution by confocal microscopy have allowed us to describe for the first time the spatio-temporal relationship of viral proteins during VSV uncoating (Fig. 11). The following events are proposed based on these data. Within 2 to 5 minutes, virions are internalized and between 5 and 10 minutes they are exposed to low pH, which can be detected by the loss of MLG fluorescence within the virion interior (Fig. 11, step A, [19]). It is well established that low pH also causes conformational changes in G protein [35], [36] which bring about the membrane fusion event [37], [38] required for VSV infection (Fig. 11, step B). During entry we found that MLG became asymmetrically distributed and localized to one side of the endosomal membrane, which we propose occurs as the virus membrane merges with the delimiting membrane of the endosome where the RNP is released into the cytoplasm (Fig. 11, step C). There was a sharp decrease in the colocalization of RNPs with MLG between 15 and 20 minutes post-entry, but since our analysis measured the physical separation of RNPs and MLG, these data suggest that the initial uncoating event would occur prior to this time. We also propose that interactions between the condensed RNP with MLG on the viral membrane maintains MLG in an organized lattice which initially restricted the quantum yield of MLG fluorescence and that after the release of RNPs into the cytoplasm this organization is lost resulting in the diffusion of MLG across the endosomal membrane and the concomitant increase in MLG fluorescence which we observed beginning approximately 15 minutes post-entry (Fig. 11, step D). As a result of uncoating, some M is released into the cytoplasm and associates with NPCs (Fig. 11, step E). By generating rVSVs encoding M-Lumio with other viral proteins fused to red, yellow, or cyan fluorescent proteins, or multiple Lumio-tagged viral proteins that are differentially labeled with Lumio-Green and Lumio-Red, it should be possible to gain further insight to the early events in VSV entry and the mechanisms of VSV uncoating.

## Materials and Methods

### Lumio Green labeling of rVSV-ML

BHK-21 cells on 100 mm plates at ∼95% confluency were infected with rVSV-ML or G-complemented ΔG-ML for 1 hour as described previously [19] and then at 4 hours post-infection (hpi) the cells were washed twice with reduced-serum Opti-MEM I (Invitrogen) and replaced with 10 ml of Opti-MEM I containing 200 nM Lumio Green (Invitrogen). The supernatant was collected 18 hpi and labeled virus was concentrated by centrifugation over a 20% sucrose cushion at 38,000 rpm in an SW41 swinging bucket rotor (Beckman) for 45 minutes. The viral pellet was resuspended on ice in 1 ml of 10% sucrose-TN buffer (10 mM Tris, pH 7.4;150 mM NaCl) and the virus was stored at −80°C. Titers were determined by standard plaque assay on BHK-21 cells and protein concentration was determined by a BCA protein assay (Pierce) according to the manufacturer's directions. Fluorescence for each virus preparation was determined as described [19].

### Expression of wild-type and dominant negative Rab proteins

BHK-21 or MDCK cells were transfected with plasmids (generous gifts from Dr. Terry Dermody, Vanderbilt University) expressing either wild-type (wt) or dominant-negative (DN) Rab proteins using either Lipofectamine (Invitrogen) for BHK cells, or Effectene (Qiagen) for MDCKs according to the manufacturers instructions. The wt and DN-Rab5 (S34N; [39], [40]), Rab7 (N125I; [41]), or Rab11 (S25N; [42], [43]) proteins were fused to eGFP in the plasmid p-eGFP (Clontech). Eighteen hours post-transfection the BHK-21 cells were infected with rVSV-wt (MOI = 10) and the MDCK cells were infected with A/Aichi/2/68 (H3N2) influenza (influenza stocks were a kind gift from Dr. Charles Russell, St. Jude Children's Research Hospital) and fixed at 8 hr (VSV), or 15 hr (influenza) post-infection. The cells were then stained for VSV N protein using monoclonal 10G4 [24], or a polyclonal influenza NP antibody conjugated to biotin (generous gift from Dr. Richard Webby, St. Jude Children's Research Hospital). Fluorescence micrographs were collected using a Zeiss Axioplan 2 microscope equipped with an HR Axiocam camera and Axiovision software. Percent inhibition was determined from approximately 90 to 150 cells in 10–20 individual fields by counting the total number of Rab-positive (eGFP-positive) cells in a field that were also VSV N- or influenza NP-positive using the formula [(1−(virus-positive cells/Rab-positive cells))*100].

### Immunofluorescence (IF) staining and confocal microscopy

rVSV-MLG infected cells were washed twice with phosphate-buffered saline, pH 7.4 (PBS) and then fixed for 15 minutes with 3% paraformaldehyde (PFA) in PBS at room temperature. The fix solution was removed and the cells washed twice with PBS containing 10 mM glycine and 0.05% sodium azide (PBS-glycine), and then permeabilized with 1% Triton X-100 in PBS-glycine at ambient temperature for 1 minute. After permeabilization, the cells were washed twice with PBS-glycine and then stained with the following antibodies as indicated in the figure legends: a) M protein using monoclonal antibody (mAb) 23H12 [24] conjugated to rhodamine, b) VSV N protein using mAb 10G4 [24] conjugated to rhodamine or after incubation with a secondary goat anti-mouse antibody conjugated to Alexa Fluor-633, c) Nup62 using mAb #610497 (BD Biosciences), d) mannose 6 phosphate receptor (M-6-P-R) using mAb #MA1-066 (Affinity BioReagents), e) LAMP-1 using an anti-LAMP-1 mAb (Developmental Studies Hybridoma Bank at the University of Iowa), f) EEA1 using a polyclonal antibody (#2411, Cell Signaling Technology), g) Rab11 using a polyclonal antibody (#71-5300, Zymed Laboratories), or h) microtubules using anti-α tubulin mAb (#A-11126, Molecular Probes). The unconjugated primary antibodies were detected with goat anti-mouse, or goat anti-rabbit secondary antibody conjugated to rhodamine (Jackson Research Laboratories), or for anti-Nup62, goat anti-mouse labeled with Zenon IgG2b Alexa Fluor-647 (#A-21242 Invitrogen; Molecular Probes). Phalloidin conjugated to Texas Red-X (Invitrogen; Molecular Probes) was used to stain actin. The distribution of the indicated proteins was examined using laser scanning confocal microscopy (Zeiss LSM 510 and AIM software version 3.2) with the multi-track setting and 488nm, 543nm, or 633nm laser excitation in 1 micron optical sections. Percent colocalization of M and N protein was accomplished using the Profile function of the Zeiss LSM Physiology software package by counting the number of N protein puncta that colocalized with M in a total of 50 cells from three different experiments. Colocalization of MLG with the endosomal markers was quantified by selecting individual cells (n =  5) and using the colocalization function of the LSM Physiology software. Colocalization of MLG with endosomal markers was determined from a 1 micron optical slice near the center plane of the cell. For colocalization analysis, the threshold settings for both green and red pixels were set at 100.

### Live-cell entry assay

BHK-21 cells plated onto 35 mm glass bottomed culture dishes (MatTek Corporation) were washed twice with ice-cold Opti-MEM I (Invitrogen) and then placed at 4°C for 15 minutes. Lumio-labeled virus (MOI 50) and human transferrin conjugated to Texas Red (50 µg) were adsorbed in 0.1 ml ice-cold Opti-MEM I for 60 minutes with rocking every 15 minutes on ice. Surface binding was examined by washing the cells 3 times with ice cold PBS. The cells were placed on ice and washed once with ice-cold 100µM desferrioxamine in PBS for 5 minutes to chelate residual iron. The cells were then washed once with ice-cold acid wash buffer (100mM sodium acetate, 50mM NaCl, pH 5.5) for 5 minutes to release transferrin-TR from transferrin receptors on the cell surface. After the acid washing the cells were washed 3 times with PBS and then incubated for 5 minutes in Opti-MEM I at 37°C. For MLG trafficking without acid washing, the ice-cold inoculum was replaced with Opti-MEM I warmed to 37°C and the cells were incubated for the times indicated. MLG and Tfn-TR were imaged in live (non-fixed) cells on a Zeiss LSM 510 laser scanning confocal microscopy using a heated stage set to 37°C and an objective heating collar using alternating 488nm and 543nm laser excitation in multi-track mode. The images were collected using identical detector gain and offset settings for each time point. The non-entry time point (t-0) was examined following addition of 2 ml of ice-cold Opti-MEM I on a stage at ambient temperature. To reduce problems of sample photobleaching, separate plates were examined for each time point.

### Cell fractionation assay

The fractionation protocol was essentially that described previously by others [44], with the following modification for infection with VSV. BHK-21 cells plated in 35 mm dishes were washed twice with ice-cold Opti-MEM I (Invitrogen) and then placed at 4°C for 10 minutes. rVSV-wt (MOI 50) was adsorbed in 0.3 ml ice-cold Opti-MEM I for 90 minutes with rocking every 15 minutes on ice. The inoculum was removed and cells were washed twice with ice-cold Opti-MEM I and then twice with ice-cold PBS. The cells were then either harvested immediately by incubation with 1 ml ice-cold MES buffer (150 mM NaCl with 25 mM MES, pH 6.5) for approximately 10 minutes (until the cells could be removed from the plate by pipetting), or were incubated for the times indicated in Opti-MEM I at 37°C. After incubation at 37°C the cells were quickly cooled by the addition of ice-cold PBS and then incubation for 10 minutes in ice-cold MES buffer after which the cell suspensions were transferred into a 1.5 ml microfuge tube on ice. All subsequent steps were performed on ice and with a microcentrifuge cooled to 4°C. Cells were disrupted using a 1 ml syringe fitted with a 25-gauge needle by forcing the cell suspension rapidly through the needle 20 times. Cell disruption was assessed via trypan blue staining, which showed >95% of the cells were trypan blue permeable after the treatment. The cell suspension was then centrifuged at 1000×*g* for 10 minutes. The supernatant was transferred to a clean tube on ice and the supernatant fraction was centrifuged again at 1000×*g*, to remove residual pelletable material. The first 1000×*g* pellet was kept on ice. The supernatant was transferred to a new tube and then spun at 16,000×g for 10 minutes. The pellet from the 16,000×g spin (P16) was washed once with ice-cold MES buffer, repelleted and then resuspended in SDS-PAGE sample buffer. The supernatant (S16) was precipitated with 10% trichloroacetic acid (TCA) and the pellet resuspended in SDS-PAGE sample buffer. The pellet from the initial 1000×*g* spin was washed once with ice-cold MES buffer, respun and then the pellet was resuspended in NDG buffer (1% Nonidet-40; 0.5% deoxycholate; 10% glycerol; 137 mM NaCl and 20 mM Tris, pH 8.0). After incubation on ice for 2 minutes the suspension was centrifuged at 16,000×*g* for 10 minutes. The pellet (NDG pellet) was washed once in NDG buffer, repelleted and then resuspended in SDS-PAGE sample buffer. The supernatant (NDG supt) was TCA precipitated and suspended in SDS-PAGE sample buffer. Fractions were electrophoresed on a 9% acrylamide gel containing SDS, transferred to Immobilon membrane and processed for immunoblot detection using the following antibodies with detection using the Pierce Dura-West Detection Reagent as described by the manufacturer. N and M proteins in the relevant fractions were quantified using Image J after scanning films and importing the images into Photoshop (Adobe) as .TIFFs. Antibodies used were a) polyclonal anti-VSV (#4006-F; Whitt lab), b) anti-Nup62 (mAb #610497; BD Biosciences), c) anti-EEA1 (polyclonal antibody #2411; Cell Signaling Technology), d) anti-Rab11 (polyclonal antibody #71-5300; Zymed Laboratories), e) anti-α tubulin (mAb #A-11126; Molecular Probes), and f) anti-mitochondrial membrane protein OxPhos Complex-I 39 kDa (CI-39; mAb #A21344; Molecular Probes).

### Entry inhibition and synchronized fusion assays

To prevent the acidification of endosomes we used either the proton ATPase inhibitor bafilomycin A1 as described previously [19], or the lysosomotropic reagent NH_4_Cl. For NH_4_Cl treatment used to synchronize fusion of virions with endosomal membranes, BHK-21 cells were washed twice with PBS and then washed twice with PBS containing 100mM NH_4_Cl. Virus inocula, either with or without 50 µg of transferrin-TR, were adsorbed in serum-free DMEM (SF-DMEM) containing 100mM NH_4_Cl for 60 or 90 minutes. After adsorption the cells were washed with PBS four times to remove the NH_4_Cl and then either fixed immediately (t-0) with 3% paraformaldehyde in PBS, pH 7.4, or after 60 minutes (t-60) following the addition of 2 ml of SF-DMEM containing 10µg/ml cycloheximide at 37°C. Synchronized fusion assays using the cytoskeletal inhibitors cytochalasin D or nocodazole were performed as described above except virus was adsorbed for 60 minutes in SF-DMEM with 100mM NH_4_Cl and the medium was replaced with SF-DMEM containing 10µM of the cytoskeletal inhibitor and 100mM NH_4_Cl for 30 minutes. The NH_4_Cl was then washed out as described above except that the cytoskeletal inhibitors remained for the time indicated and then the cells were fixed with 3% PFA and prepared for IF.
